# Bacterial Etiology and Antibiotic Resistance Patterns in Neonatal Sepsis in Tehran during 2006-2014

**Published:** 2017-10-01

**Authors:** Fatemeh Haj Ebrahim Tehrani, Mohammad Moradi, Narjes Ghorbani

**Affiliations:** 1 *Dept. of Neonatology, Shahed Medical Faculty, Shahed University, Tehran, Iran*; 2 *Students Research Committee of Shahed Medical Faculty, Shahed University, Tehran, Iran*; 3 *Shahed Medical Faculty, Shahed University, Tehran, Iran*

**Keywords:** Neonatal Sepsis, *Antibiotics*, Antibiogram, Resistance

## Abstract

**Background & Objective:**

Neonatal sepsis is one of the leading causes of mortality in neonatology wards. The aim of this study was to assess sepsis pathogens and antibacterial resistance patterns in a teaching hospital during seven years in Tehran, Iran.

**Methods:**

In this retrospective study, all neonates suspected to sepsis and fulfilling the sepsis criteria admitted to NICU ward of Mustafa Khomeini Hospital, Tehran, Iran during 2007 to 2014 were included. Demographic information, blood test results, blood culture results of neonates and antibiogram findings were extracted from their documents. Data was analyzed using SPSS 15.

**Results:**

Ninety neonates with positive culture test were included. Fifty-three were male (58.9%). Thirty neonates were delivered vaginally (33.3%) and 60 caesarean section (66.7%). Most bacterial growths in culture were *Staphylococcus aureus* and *E. coli*. The rates of resistance for antibiotics like ceftriaxone, cefotaxim and gentamycin were 5%, 30% and 15%, correspondingly. There were 15 cases (16.7%) with resistance to imipenem.

**Conclusion:**

Antibacterial resistance patterns vary in different parts of the world and even within a country, therefore assessing resistance patterns in a region is of great importance for proper management and treatment. Our findings might help physicians for proper selection of antibiotics for treatment of neonatal sepsis.

## Introduction

Neonatal septicemia is microbial growth in the blood of a neonate accompanied with clinical manifestations. Septicemia is one of the most common neonatal diseases worldwide (1-3). One of the reasons of neonatal septicemia is undevelopment of immune system in this group (4, 5). Sepsis is more prevalent in premature neonates and those with low birth weight (6). It may reach 30% in very low birth weight under intensive care (7). Dangerous complications include meningitis and brain abscess leading to severe morbidity and mortality. While mortality due to meningitis has been decreased in the recent years (50% in 1972 to less than 10% in 1977), neurologic complications are of major problems of these neonates (8-10).

Neonatal sepsis is manifested as early onset or late onset. Early onset septicemia initiates at first 96 hours of life (4 days) with a high mortality rate, usually originated from mother genitalia microorganisms. Different reasons including delivery type, premature delivery, premature rupture of membrane, uterine inertia, fever and maternal infection increase the probability of sepsis (11). 

The most important factor in decreasing neonatal mortality and its comorbidities is prompt diagnosis and treatment. Definite diagnosis of sepsis is based on bacterial culture methods, which may be time consuming and sometimes leading to some complications (12, 13).

Age and immune status of neonate are very important to define the response of body to sepsis. Causative microorganisms vary in different regions including *Streptococcus *group B*, Escherichia coli, Klebsiella, Listeria monocytogenes *and* Haemophilus influenza*. Therefore, epidemiologic studies are of great importance in each region. In premature neonates, coagulase negative *Staphylococcus* is more prevalent. *Candida *spp. should be also considered. In older children with fever and intact immune system, most common pathogens include *S. pneumonia, L monocytogenes *and* H. influenza*. In children under five years, *Salmonella, Staphylococcus aureus *and* Streptococcus *group A are causative pathogens. Children with pyelonephritis often suffer gram negative bacteremia like *E. coli*.

Immunocompromised children (like those without spleen, sickle cell anemia, neutropenia, malignancy, AIDS or foreign body aspiration) are infected with a wide spectrum of microorganisms and are more prone to septicemia; fungal infection may also occur in these children (14-17). 

In some patients, bacteremia is accompanied with local infections (like pyelonephritis, pneumonia, cellulitis, osteomyelitis, endocarditis and meningitis). In such situations, when there is a suspicion to bacteremia, blood culture is performed to definite the diagnosis and intravenous wide spectrum antibiotics are administered (18). 

Septic shock is caused by body reaction to microbial products such as peptidoglycans of Gram-positive bacteria and lipopolysaccharides of Gram-negative organisms. Lipopolysaccharides, peptidoglycans and lipoproteins are introduced to macrophages by toll-like receptors, which induce the immune cascade against the microorganisms (19). 

Early onset sepsis is a severe multiorgan disease manifested with pulmonary failure, shock, meningitis, DIC (Disseminated Intravenous Coagulation) and acute tubular necrosis. Late onset sepsis is usually occurred in healthy neonates discharged from the neonatal ward (20).

Hospital sepsis (until day eight of discharge) is mostly occurred in preterm children in neonatal intensive care unit. The infection is occurred mostly due to NICU infection with multidrug resistant species. 

When the neonate is febrile and a toxic appearance (tachycardia, agitation, lethargy, hyperventilation and decreased peripheral perfusion), septicemia is strongly suspected. History taking and physical examination may reveal the disease origin (21). Primary symptoms resemble many other infections. Shivering, hyperventilation, tachycardia, hypotension and reduced peripheral vascular resistance are some signs and symptoms. Skin rashes like petechial and purpuric lesions are pathogenic for meningococcemia. *Pseudomonas aeruginosa* has specific skin lesions called the Bull’s eye lesions. DIC may also occur with purpura and hemorrhage from blood sampling sites. Hypotension may lead to peripheral gangrene, anuric renal failure and lactate acidosis (22). 

Gender might have an effect on the incidence of sepsis in newborns, but the results of different performed studies are controversial. For instance, a study in Mofid Hospital showed that male to female ratio was 1.7 (23). However, in a study performed on 5447 neonates with sepsis, there was no meaningful difference between genders (24). 

As mentioned above, preterm neonates are at risk of acquiring neonatal sepsis. For example, sepsis was not common in preterm neonates (1.5% prevalence of sepsis in preterm neonates), but 37% of them died, indicating that preterm is still an important risk factor of mortality (24). 

The aim of this study was to assess bacterial agents and antibiotics resistance patterns in neonatal sepsis of Mostafa Khomeini Hospital of Tehran, Iran.

## Material and Methods

This retrospective cross-sectional study was performed during 2006 and 2014 in Mostafa Khomeini Hospital, Tehran, Iran. All neonates suspected to sepsis confirmed by positive results for blood culture were enrolled in this study. Ninety patients fulfilled the inclusion criteria. Patients’ information was extracted from hospital documents. Data including sepsis type, age, gender, type of cultured pathogen, delivery method, antibiogram, mortality rate, premature rupture of membrane and birth body weight were recorded. Antibiogram was performed using antibiotics disks on Muller-Hinton plates. Results were read 48 hours later by a professional technician. Assessment was performed by measuring the halo diameter on millimeter and using special antibiogram charts. Sepsis in first 96 hours after birth was considered as primary sepsis and after it as secondary sepsis.

Ethical aspects were observed and informed consent was taken from subjects’ parents.

Data were recorded in researcher made forms and then entered SPSS 16 (SPSS Inc., Chicago, IL, USA). Descriptive and analytic analyses were performed using mean, standard deviation, Chi-2 and *t* test.

## Results

 Overall, 90 neonates had positive results for blood culture considered as sepsis. Fifty-nine (65.6%) neonates had primary sepsis and 31 (34.4) had late sepsis. Regarding birth body weight, one neonate weighted below 1000 gr, one between 1000 and 1500 gr, 41 between 1500 and 2500 and 47 more than 2500 gr. Overall, 47.8% weighted below 2500 gr. Fifty-three were male. 

Regarding the etiologic pathogen, 27 neonates (30%) had sepsis due to coagulase negative *Staphylococcus*. Twenty-four (26.7%) *S. aurous*, 12 (13.3%) *E. coli*, 10 (11.1%) *Klebsiella*, 6 (6.7%) *P. aeruginosa*, 7 (7.8%) *S. epidermidis*, three (3.3%) *Enterobacter* and one (1.1%) group D* Streptococcus*. Thirty (33.3%) neonates were delivered by normal vaginal delivery and 60 (66.6%) by cesarean section. Fifty-six neonates (62.2%) were delivered on maturity and 34 (37.8%) premature. Overall, pathogens isolated from 88 (97.8%) neonates had resistance to at least one antibiotic examined and two neonates had no resistance to any of the examined antibiotics. Six neonates (6.7%) died due to sepsis and 84 (93.3%) discharged. Forty-six neonates (51.1%) had premature rupture of membrane and 44 (48.9%) did not.

Using Chi-2 test, there was no significant and meaningful difference between pathologic agents in the two groups of early and late sepsis. Pathologic agents in the two groups of early and late sepsis are shown in Table 1.

**Table 1:** Pathologic Agents in the Study Neonates in Early and Late Sepsis

Bacteria	Early Sepsis	Late Sepsis
Coagulase negative* Staphylococcus*	22	5
*Staphylococcus aurous*	13	11
*Klebsiella*	5	5
*Pseudomona aeruginosa*	3	3
*Staphylococcus epidermidis*	6	1
*Enterobacter*	2	1
Group* D streptococcus*	1	0
*Escherichia coli*	7	5

Regarding age, there was no meaningful difference in terms of pathologic agent between male and female neonates using Chi-2 test for each bacterium separately (P=0.509). Regarding delivery type, there was no meaningful difference in terms of pathologic agent between NVD (Normal Vaginal Delivery) and C-section using Chi-2 test (P=0.282). However, there was no meaningful difference in terms of pathologic agent between neonates with and without premature rupture of membrane using 

Chi-2 test (P=0.218). There was no meaningful difference in terms of pathologic agent between neonates with different weights (P=0.218). In addition, mortality rate did not differ significantly between sepsis caused by different bacteria. Despite the fact, there was a meaningful difference regarding pathologic agents between mature and premature neonates (P=0.03); for instance, E. coli was only detected in mature neonates and no case of E. coli infection in premature ones. Antibiotic resistance patterns of different bacteria are depicted in Table 2.

**Table 2:** Antibiotic Resistance Patterns of Different Bacteria

	Coagulase Negative* Staphylococcus*	*S. aurous*	*Klebsiella*	*P. aeruginosa*	*S. epidermidis*	*Enterobacter*	*Group D streptococcus*	*E. coli*	Total
Co-amoxyclave	2	1	8	0	0	2	0	8	21
Cefexime	0	0	0	0	0	0	0	1	1
Piperacillin	0	3	0	0	3	0	0	1	7
Cefotaxim	11	7	0	1	0	0	0	8	27
Imipenem	10	0	0	0	4	0	0	1	15
Ticarcillin	0	0	0	0	0	0	0	1	1
Cotrimoxazole	0	6	0	0	0	0	1	1	8
Ampicillin	15	8	3	4	5	1	0	4	40
Gentamycin	3	1	5	4	0	0	0	1	14
Nitrofurantoin	0	0	5	0	0	0	0	1	6
Amikacin	0	0	5	3	0	0	0	4	12
Oxacillin	17	14	1	0	5	0	0	1	38
Clindamycin	16	13	0	2	0	0	0	1	32
Ciprofloxacin	4	8	2	1	0	0	0	2	17
Ceftrizaxon	3	1	0	0	0	0	0	0	4
Carbenicillin	0	0	2	3	0	0	1	0	6
Cefalexin	0	1	0	0	0	0	0	0	1
Ceftriaxone	0	1	4	0	0	0	0	0	5
Cefalotin	1	7	8	1	0	3	1	7	28
Tetracycline	6	0	0	0	0	0	0	4	10
Rifampin	0	2	0	0	0	0	1	1	3
Methicillin	1	3	0	0	0	0	0	0	4
Erythromycin	14	11	0	1	0	0	0	1	27
Chloramphenicol	3	3	0		0	0	0	0	6
Penicillin G	25	20	0	1	3	0	1	1	51
Neomycin	0	1	0	0	2	0	0	0	3
Total	116	97	43	21	23	6	4	43	388

## Discussion

Sepsis is one of the most important causes of neonatal mortality. Around 2% of fetus have intrauterine infection and 10% in the first month of their life. Of 90 patients with positive results for blood culture, 53 were male and 37 females (male to female ratio=1.43). However, in a study performed on 5447 neonates with sepsis, there was no meaningful difference between genders (24). Of total patients, 65% had early sepsis and the other had late one. Early sepsis is more common in developed countries (25). Regarding maturity, 62% of neonates were term and 38% preterm. Sepsis was not common in preterm neonates (1.5% prevalence of sepsis in preterm neonates), but 37% of them died. Indicating that preterm is still an important risk factor of mortality (24). In total, 33% of neonatal sepsis in our study were born by NVD and 66% by C-section.

Several studies had been performed on sepsis in developing countries, in which Gram-negative bacteria had been the main causative agents. For instance, *Klebsiella* and *Pseudomonas* were the main causative agents in India for neonatal sepsis (26). Microorganisms involved in sepsis differ in neonates of different regions. Stoll reported Group B *streptococcus* as the most common agent in the America, but in non-American countries, *Staphylococcus aurous* was the most common agent (27). 

The novelty of this study was assessing resistance patterns to different antibiotics, which help proper selection of treatment regimen. The least resistance in our study was to Cefixime, Ticarcillin and cephalexin with only one isolate being resistance. Therefore, it is concluded that these three antibiotics may be used with good efficacy in the treatment of neonatal sepsis. 

On the other hand, the most resistance in our study isolates was against Ampicillin and penicillin G with 40 and 51 isolates. In this study, we assessed resistance patterns against 26 antibiotics, which is a strong point of the current study. Resistance patterns of this study should be used for selecting antibiotics regimen for treatment of neonatal sepsis.

At present, sepsis treatment in our country is mostly based on foreign investigations in other regions, which cannot be truly generalized to our settings due to social, cultural and geographical differences. This study could shed light for experimental treatment of neonatal sepsis in our country for more accurate and effective treatment. Larger studies in different health care centers of the country can provide more comprehensive information. In addition, using different culture media and repeating the examinations when necessary or analyzing the cerebral fluid in case of treatment failure may improve diagnosis and treatment probability. 

## Conflict of interests

All authors contributed equally in preparing the manuscript

## References

[1] Charles MV, Srirangaraj S, Kali A (2015). Neonatal sepsis caused by Shewanella algae: A case report. Australas Med J.

[2] Hossain S, Shah PS, Ye XY, Darlow BA, Lee SK, Lui K (2015). Outcome comparison of very preterm infants cared for in the neonatal intensive care units in Australia and New Zealand and in Canada. J Paediatr Child Health.

[3] Obiero CW, Seale AC, Berkley JA (2015). Empiric treatment of neonatal sepsis in developing countries. Pediatr Infect Dis J.

[4] Sobouti B, Fallah S, Mobayen M, Noorbakhsh S, Ghavami Y (2014 ). Colonization of Mycoplasma hominis and Ureaplasma urealyticum in pregnant women and their transmission to offspring. Iran J Microbiol.

[5] Zareifar S, Pishva N, Farahmandfar M, Benaei S, Cohan N (2014 ). Prevalence of G6PD Deficiency in Neonatal Sepsis in Iran. Iran J Pediatr.

[6] Ballén V, Sáez E, Benmessaoud R, Houssain T, Alami H (2015 ). First report of a Klebsiella pneumoniae ST466 strain causing neonatal sepsis harbouring the blaCTX-M-15 gene in Rabat, Morocco. FEMS Microbiol Lett.

[7] Sharma D, Shastri S (2015 ). Lactoferrin and neonatology - role in neonatal sepsis and necrotizing enterocolitis: present, past and future. J Matern Fetal Neonatal Med.

[8] Fang DH, Fan CH, Li J, An Q, Yao H, Ji Q, Niu G (2015). Ratios of CD64 expressed on neutrophils, monocytes, and lymphocytes may be a novel method for diagnosis ofneonatal sepsis. J Infect Dev Ctries.

[9] Marwah P, Chawla D, Chander J, Guglani V, Marwah A (2015 ). Bacteriological Profile of Neonatal Sepsis in a Tertiary-care Hospital of Northern India. Indian Pediatr.

[10] Cotten CM (2015 ). Antibiotic Stewardship: Reassessment of Guidelines for Management of Neonatal Sepsis. Clin Perinatol.

[11] Capasso L, Borrelli A, Cerullo J, Pisanti R, Figliuolo C (19). Role of immunoglobulins in neonatal sepsis. Transl Med UniSa.

[12] Kirkham EN, Heaton PA, Paul SP (2014 ). At a glance: neonatal sepsis in the community. J Fam Health Care.

[13] Delanghe JR, Speeckaert MM (2015). Translational research and biomarkers in neonatal sepsis. Clin Chim Acta.

[14] Sharma D, Kumar C, Pandita A, Pratap OT, Dasi T, Murki S (2015). Bacteriological profile and clinical predictors of ESBL neonatal sepsis. J Matern Fetal Neonatal Med.

[15] Trend S, Strunk T, Hibbert J, Kok CH, Zhang G, Doherty DA, Richmond P (2015 ). Antimicrobial protein and Peptide concentrations and activity in human breast milk consumed by preterm infants at risk of late-onset neonatal sepsis. PLoS One.

[16] Machado JR, Soave DF, da Silva MV, de Menezes LB, Etchebehere RM, Monteiro ML (2014). Neonatal sepsis and inflammatory mediators. Mediators Inflamm.

[17] Zea-Vera A, Ochoa TJ (2015). Challenges in the diagnosis and management of neonatal sepsis. J Trop Pediatr.

[18] Ng PC1, Ma TP1, Lam HS1 (2015 ). The use of laboratory biomarkers for surveillance, diagnosis and prediction of clinical outcomes in neonatal sepsis and necrotising enterocolitis. Arch Dis Child Fetal Neonatal Ed.

[19] Ayazi P, Mahyar A, Daneshi MM, Jahanihashemi H, Esmailzadehha N, Mosaferirad N (2014). Comparison of serum IL-1beta and C reactive protein levels in early diagnosis and management of neonatal sepsis. Infez Med.

[20] Giménez M, Sanfeliu I, Sierra M, Dopico E, Juncosa T, Andreu A (2014). [Group B streptococcal early-onset neonatal sepsis in the area of Barcelona (2004-2010). Analysis of missed opportunities for prevention.]. Enferm Infecc Microbiol Clin.

[21] Hedegaard SS, Wisborg K, Hvas AM (2015). Diagnostic utility of biomarkers for neonatal sepsis--a systematic review. Infect Dis (Lond).

[22] Amid Mohammad Hosein, Assessing sepsis, meningitis cases in in neonates admitted in Mofid Hospital (1998). Research in Medicine Journal.

[23] Stoll BJ1, Hansen N, Fanaroff AA, Wright LL, Carlo WA, Ehrenkranz RA, Lemons JA, Donovan EF, Stark AR, Tyson JE, Oh W, Bauer CR, Korones SB, Shankaran S, Laptook AR, Stevenson DK, Papile LA, Poole WK (25). Changes in pathogens causing early-onset sepsis in very-low-birth-weight infants. N Engl J Med.

[24] Palazzi DL, Klein JO, Baker CJ (2005). Bacterial sepsis and meningitis in Remington. Clin Infect Dis.

[25] Smith PB1, Cotten CM, Garges HP, Tiffany KF, Lenfestey RW, Moody MA, Li JS, Benjamin DK Jr A comparison of neonatal Gram-negative rod and Gram-positive cocci meningitis. J Perinatol. 2006.

[26] S Vergnano, M Sharland, P Kazembe, C Mwansambo, and P Heath (2005). Neonatal sepsis: an international perspective. Arch Dis Child Fetal Neonatal Ed.

[27] Stoll BJ1 (1997). The global impact of neonatal infection. Clin Perinatol.

